# Efficacy of ranibizumab for the treatment of diabetic retinopathy: A protocol for systematic review of randomized controlled trial

**DOI:** 10.1097/MD.0000000000015409

**Published:** 2019-04-26

**Authors:** Yong-bo Ren, Xing-jie Su, Yan-xiu Qi, He-qun Luan, Qi Sun

**Affiliations:** Department of Ophthalmology, First Affiliated Hospital of Jiamusi University, Jiamusi, China.

**Keywords:** diabetic retinopathy, efficacy, ranibizumab, safety, systematic review

## Abstract

**Background:**

Previous clinical trials have reported that ranibizumab can be used to treat diabetic retinopathy (DR) effectively. However, no study has been conducted to evaluate its efficacy for patients with DR systematically. Thus, this study will specifically and systematically assess the efficacy and safety of ranibizumab for DR.

**Methods:**

Cochrane Library, EMBASE, PUBMED, Web of Science, Google Scholar, Cumulative Index to Nursing and Allied Health Literature, China National Knowledge Infrastructure, and Chinese Biomedical Literature Database will be searched from inceptions to the March 20, 2019 for studies related to the topic. This study will only consider publicly released randomized controlled trials for evaluating the effect and safety of ranibizumab for DR. No language restrictions will be imposed for all databases search. Methodological quality of each included trial will be assessed by Cochrane risk of bias tool. Statistical analysis will be performed by Stata 12.0 software.

**Results:**

This study will provide recent summary evidence of ranibizumab for DR. Primary outcomes include percentages with retinopathy improvement, and cumulative probabilities for retinopathy worsening. Secondary outcome consist of visual function, best-corrected visual acuities, central subfield thickness, total macular volume, peripheral visual field loss, retinal neovascularization, and adverse events.

**Conclusion:**

The findings of this study may provide theoretical basis for clinical practice refer and may benefit more patients with DR.

## 1 Introduction

Diabetic retinopathy (DR) is one of the most common complications in patients with diabetes mellitus (DM),^[1–3]^ which account for about 40% of DM over 40 years of age.^[4–5]^ Previous study found that over 75% of patients with DM for more than 20 years will likely develop DR.^[6]^ It is also the leading cause of impaired vision and even blindness in patients with DM,^[7]^ and contributes 4.8% of 37 million cases of blindness worldwide.^[8]^

Lots of clinical studies have reported that ranibizumab can be widely and effectively utilized to treat patients with DR with promising efficacy.^[9–27]^ However, no study has specifically addressed the efficacy and safety of ranibizumab for patients with DR. In this study, we specifically focused on assessing the efficacy and safety of ranibizumab for DR systematically.

## 2 Methods

### 2.1 PROSPERO registration

This study has been registered on PROSPERO with registration number PROSPERO CRD42019127058.

### 2.2 Eligibility criteria

#### 2.2.1 Types of trials

This study will consider randomized controlled trials (RCTs) of ranibizumab as a way to improve DR without language restrictions. In addition to the RCTs, any other types of trials will all be excluded.

#### 2.2.2 Types of patients

All the patients who meet the relevant diagnostic criteria for DR will be fully considered without any restrictions of race, age, gender, and case source.

#### 2.2.3 Types of interventions

This study will utilize ranibizumab as an only intervention in the experimental group. However, in the control group, patients can receive any treatments.

#### 2.2.4 Types of outcome measurements

##### 2.2.4.1 Primary outcomes

- Percentages with retinopathy improvement;
- Cumulative probabilities for retinopathy worsening.

##### 2.2.4.2 Secondary outcomes

- Visual function (as measured by National Eye Institute Visual Function Questionnaire-25 or other scales);
- Best-corrected visual acuities;
- Central subfield thickness;
- Total macular volume;
- Peripheral visual field loss;
- Retinal neovascularization;
- Any adverse events.

### 2.3 Search methods for the identification of eligible trials

Cochrane Library, EMBASE, PUBMED, Web of Science, Google Scholar, Cumulative Index to Nursing, and Allied Health Literature, China National Knowledge Infrastructure, and Chinese Biomedical Literature Database will be searched from inceptions to the March 20, 2019. The search language will not be limited. We will also search non-electronic papers manually, and reference lists of eligible trials, as well as relevant reviews. The search strategy with details is demonstrated in Table 1. In addition, similar search strategies will also be utilized to any other databases.

**Table 1:**
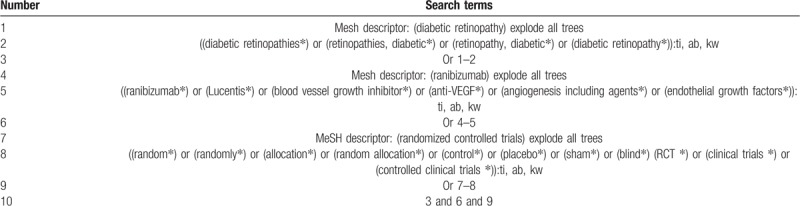
Search strategy for Cochrane Library.

### 2.4 Study selection

Two investigators will scan the titles and abstracts independently to exclude any irrelevant studies. Then, full texts will be further assessed according to the predefined eligibility criteria. Any disputes regarding the study selection between 2 investigators will be resolved through discussion with the assistance of a third investigator. The whole process of study selection is abided to the guidelines of the Preferred Reporting Items for Systematic Reviews and Meta-Analyses (PRISMA)^[28]^ and PRISMA-Protocol guidelines.^[29–30]^ Its results will be presented in a PRISMA flow chart with clearly reasons of exclusion and inclusion at each stage.

### 2.5 Data extraction and management

Two investigators will independently accomplish data extraction according to the previous designed data extraction form. A third investigator will be invited to solve any disputes between 2 investigators. It will collect and extract following information: including first author, time of publication, location, study setting, diagnostic criteria, eligibility criteria; age, gender, race, number of patients in each group; methods of randomization, concealment, and blinding; intervention details, drug, dosage, frequency, duration; all outcome indicators, and adverse events.

### 2.6 Missing data management

Whenever there is missing data or incomplete information, we will contact the primary authors to request those data by email or telephone. If those data is still not available, we will discuss its possible effects.

### 2.7 Risk of bias assessment

Risk of bias assessment will be completed by 2 investigators independently according to the standard criteria of Cochrane Handbook of Systematic Review of Interventions. It includes 7 items, and each item is further divided into 3 levels of high, unclear, and low risk of bias. Any disagreements regarding the risk of bias assessment between 2 investigators will be resolved by a third investigator through discussion.

### 2.8 Statistical analysis

Binary outcome data will be presented as risk ratio and 95% confidence intervals (CIs). Continuity outcome data will be represented as mean difference, or standardized mean difference and 95% CIs.

Heterogeneity among eligible RCTs will be identified by *I*^2^ test. The value of *I*^2^ ≤ 50% indicates acceptable heterogeneity. Otherwise, the value of *I*^2^ > 50% indicates significant heterogeneity. If there is acceptable heterogeneity, a fixed-effect model will be applied, and we will perform meta-analysis. On the other hand, if there is significant heterogeneity, a random-effect model will be used, and group analysis will be carried out to identify any potentials causes for the high heterogeneity.

### 2.9 Additional analysis

#### 2.9.1 Subgroup analysis

Subgroup analysis will be carried out based on the different characteristics, interventions, controls, and outcomes to identify any potential factors that may result in the high heterogeneity.

#### 2.9.2 Sensitivity analysis

Sensitivity analysis will be conducted to examine the stability for the analysis results by removing high risk of bias of eligible trials.

#### 2.9.3 Reporting bias

Publication bias will be checked by Funnel plot and Egger regression test when sufficient RCTs are eligible, normally more than 10 studies.

## 3 Discussion

This systematic review is the first study to specifically assess the efficacy and safety of ranibizumab for patients with DR. It will comprehensively search a variety of databases without any language restrictions. Its results will supply a detailed summary of the up-to-date evidence relevant of ranibizumab for patients with DR. The evidence may be helpful to either the clinical practice and patients, or the health policy makers regarding the specific use of ranibizumab for patients with DR.

## Author contributions

**Conceptualization:** Yong-bo Ren, Xing-jie Su, Yan-xiu Qi, He-qun Luan.

**Data curation:** Yong-bo Ren, Xing-jie Su, Qi Sun.

**Formal analysis:** Yong-bo Ren, Yan-xiu Qi, He-qun Luan, Qi Sun.

**Funding acquisition:** Yong-bo Ren.

**Investigation:** Xing-jie Su.

**Methodology:** Xing-jie Su, Yan-xiu Qi, He-qun Luan, Qi Sun.

**Project administration:** Xing-jie Su.

**Resources:** Yong-bo Ren, Yan-xiu Qi, He-qun Luan, Qi Sun.

**Software:** He-qun Luan, Qi Sun.

**Supervision:** Xing-jie Su, Yan-xiu Qi.

**Validation:** Yong-bo Ren, He-qun Luan, Qi Sun.

**Visualization:** Yong-bo Ren, Xing-jie Su, Qi Sun.

**Writing - original draft:** Yong-bo Ren, Xing-jie Su, Yan-xiu Qi, He-qun Luan, Qi Sun.

**Writing - review & editing:** Yong-bo Ren, Xing-jie Su, Yan-xiu Qi, Qi Sun
